# First Detection of Avian Lineage H7N2 in *Felis catus*

**DOI:** 10.1128/genomeA.00457-17

**Published:** 2017-06-08

**Authors:** Sandra P. Newbury, Francine Cigel, Mary Lea Killian, Christian M. Leutenegger, M. Alexis Seguin, Beate Crossley, Robin Brennen, David L. Suarez, Mia Torchetti, Kathy Toohey-Kurth

**Affiliations:** a School of Veterinary Medicine, University of Wisconsin–Madison, Madison, Wisconsin, USA; b Wisconsin Veterinary Diagnostic Laboratory, Madison, Wisconsin, USA; c National Veterinary Services Laboratories, Veterinary Services, U.S. Department of Agriculture, Ames, Iowa, USA; d IDEXX Laboratories, Inc., West Sacramento, California, USA; e California Animal Health and Food Safety Laboratory, University of California, Davis, Davis, California, USA; f Animal Care Centers of New York City, New York, New York, USA; g Southeast Poultry Research Laboratory, Athens, Georgia, USA

## Abstract

In December 2016, influenza A (H7N2) was first detected among cats in the New York City shelter system with subsequent widespread transmission. The sequence of the first clinical isolate, A/feline/New York/16-040082-1/2016(H7N2), and its genetic similarity to the live bird market lineage of H7N2 low-pathogenicity avian influenza are described.

## GENOME ANNOUNCEMENT

Influenza A viruses infect many mammalian and avian hosts. Determination of the subtype, pathotype, and virus lineage is a critical step when confronted with the emergence of an influenza strain in new species. Here, we report the identification of influenza A (H7N2) virus which was readily transmitted among cats in the New York City shelter population.

In November of 2016, a severely ill cat showing clinical signs of respiratory disease was euthanized in a New York City animal shelter. A specimen was sent to IDEXX Reference Laboratories for a respiratory PCR panel. An influenza A virus with an N2 subtype was detected and initially reported as presumptive H3N2 canine influenza. However, in consultation with the University of Wisconsin–Madison Shelter Medicine Program, it was recognized that the pattern of transmission among cats, as well as the notable lack of detection in dogs housed in the same facilities, required further investigation. Additional specimens were submitted to the Wisconsin Veterinary Diagnostic Lab and by IDEXX to the California Animal Health and Food Safety Laboratory. Subtype determination by gene-specific PCR and Sanger sequence analysis revealed an H7N2 influenza A virus. Specimens were analyzed simultaneously at the National Veterinary Services Laboratory, confirming North American lineage H7N2 based upon next-generation sequencing direct from the sample and from a recovered virus (1).

Genome analysis from the first clinical case (A/feline/New York/16-040082-1/2016) indicates that all eight genes are highly related to H7N2 live bird market (LBM) lineage low-pathogenic avian influenza (LPAI) viruses that were eradicated from LBM poultry in 2006. Sequence similarity of 96 to 98% at the nucleotide level (95 to 99% at the amino acid level) was closest to LBM strains from the early 2000s. The feline H7N2 viral sequence had several changes associated with increased adaptation for pathogenicity in mammals, including aspartic acid at position 701 of the PB2 protein, serine at position 66 of the PB1-F2 protein, and aspartic acid at position 30 and alanine at position 215 of the matrix 1 protein. However, these same changes were present in all of the historic H7N2 LPAI viruses of the LBM lineage and therefore are unlikely to be the result of current adaptation to cats (2–4).

Detection of avian lineage influenza A strains in cats has been previously documented. Cats have been infected by the highly pathogenic strains H5N1 and H7N7 with limited transmission (5, 6). Other low-pathogenic strains (H6N4 and H1N9) have been experimentally introduced into cats (7), but we are unaware of other avian lineage strains being transmitted with ease, as demonstrated by the spread among New York City shelter cats in late 2016 that infected several hundred cats. It is unknown how the influenza A (H7N2) virus was introduced into cats in the shelter, and further studies are ongoing.

### Accession number(s).

This whole-genome sequence of A/feline/New York/16-040082-1/2016(H7N2) has been deposited in GenBank under the accession numbers KY888121 (PB2), KY888122 (PB1), KY888123 (PA), KY888124 (HA), KY888125 (NP), KY888126 (NA), KY888127 (MP), and KY888128 (MP).
